# A Case of Effective Mepolizumab Induction Therapy for Severe Eosinophilic Granulomatosis with Polyangiitis Diagnosed by Eosinophilic Cholecystitis and Interstitial Nephritis

**DOI:** 10.1155/2021/6678893

**Published:** 2021-06-19

**Authors:** Keita Hattori, Yuri Teramachi, Yoshinori Kobayashi, Takeshi Ito, Takatoshi Morinaga, Hirohumi Tamai, Yoshihiro Yamamoto

**Affiliations:** ^1^Division of Nephrology, Department of Internal Medicine, Anjo Kosei Hospital, Anjo, Japan; ^2^Division of Nephrology, Department of Internal Medicine, Toyota Memorial Hospital, Toyota, Japan

## Abstract

A 66-year-old man with a history of bronchial asthma and sinusitis was admitted with cholecystitis and peripheral neuropathy. The histopathological findings of the gallbladder revealed necrotic vasculitis and granulomatous inflammation with marked eosinophilic infiltration. Kidney biopsy also showed marked eosinophilic infiltration in the tubulointerstitial area and eosinophilic tubulitis. He was diagnosed with eosinophilic granulomatosis with polyangiitis (EGPA) and treated with corticosteroids. However, he showed no response. Therefore, he was administered mepolizumab 300 mg, which resulted in clinical improvement, including normalization of the eosinophil and CRP levels. We herein describe the first case of successful induction therapy of EGPA using mepolizumab.

## 1. Introduction

Eosinophilic granulomatosis with polyangiitis (EGPA), also known as Churg–Strauss syndrome, is characterized by asthma, eosinophilia, fever, and necrotizing and granulomatous vasculitis [1]. It also causes multiple organ failure of the heart, kidneys, lungs, and gastrointestinal tract. EGPA occurring with eosinophilic cholecystitis and interstitial nephritis is rare; delayed diagnosis causes aggravation. The Five-Factor Score (FFS), which indicates prognostic factors of EGPA, includes gastrointestinal and renal disorders, which, when present, indicate a worse prognosis [2]. Thus, early therapeutic intervention is indispensable. The first line drug choice for induction therapy in EGPA includes corticosteroids, which, in severe cases, are combined with immunosuppressants or intravenous immunoglobulin (IVIG). To date, these medications have shown limited benefits and are accompanied by significant adverse reactions. In 2017, a double-blind randomized controlled trial (RCT) provided evidence supporting the efficacy and safety of mepolizumab in refractory or relapsing EGPA [3]. Therefore, mepolizumab can be officially considered an additional therapeutic option, with a steroid-sparing effect, for cases of relapsing or refractory EGPA. To the best of our knowledge, this is the first report of successful treatment of EGPA with induction therapy using mepolizumab. Here, we present a case of severe EGPA that was successfully treated with mepolizumab, following its diagnosis based on eosinophilic cholecystitis and interstitial nephritis.

## 2. Case Presentation

**Figure 1:**
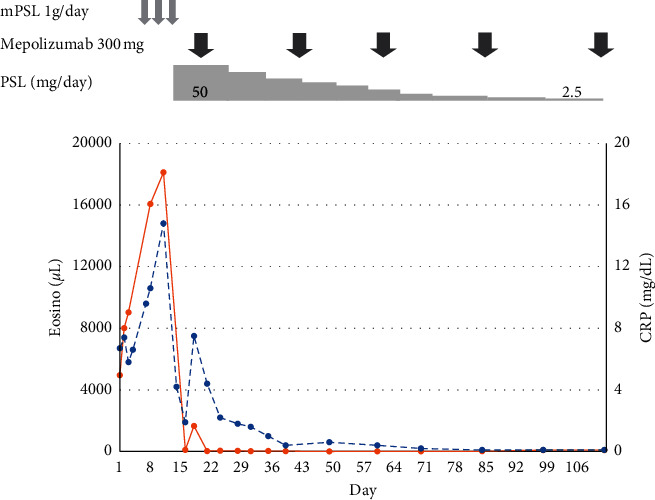
The laboratory data over the clinical course: eosinophils (eosino) and C-reactive protein (CRP). Methylprednisolone (mPSL, 1 g/day) was administered for 3 days. Prednisolone (PSL, 50 mg/day) was started on day 14 and tapered every week or fortnightly. Mepolizumab was administered on day 18.

The patient in this case is a 66-year-old Japanese man with a medical history of chronic sinusitis. At 62 years of age, he was diagnosed with asthma and subsequently treated with an inhaled corticosteroid (ICS) and long-acting *β*-agonist (LABA). He had experienced fatigue, anorexia, and a slight fever for 2 months and epigastralgia for 1 week. He visited a nearby clinic, where it was determined that the levels of his hepatic enzymes were elevated. He was diagnosed with cholecystitis and referred to our hospital, where, upon admission, he presented with a sudden gait disturbance. On physical examination, his vital signs were recorded as follows: body temperature, 37.3°C; blood pressure, 169/107 mmHg; pulse rate, 86 beats/min; and oxygen saturation, 96% in room air. A detailed physical examination revealed frontal sinus tenderness, bilateral wheezing, and purpura of approximately 1 mm in diameter bilaterally on the lower limbs. He did not have abdominal pain or signs of peritoneal irritation. Manual muscle testing (MMT: right/left) showed that pronator teres and flexor carpi radialis were 3/3, quadriceps 4/1, iliopsoas 4/3, and tibialis anterior 5/3. Biceps, triceps, and patellar tendon reflex were completely lost. Sensory disturbance was observed in both his upper and lower limbs. Laboratory testing of blood counts revealed marked eosinophilia (white blood cells (WBC): 14,300/*µ*L and eosinophils: 8,000/*µ*L), polyclonal hyperimmunoglobulinemia (IgG: 2,209 mg/dL, IgG4: 1,220 mg/dL, and IgE: 2,482 IU/dL), and an inflammatory response (C-reactive protein (CRP): 7.40 mg/dL) (Table 1 and Figure 1).

**Figure 2:**
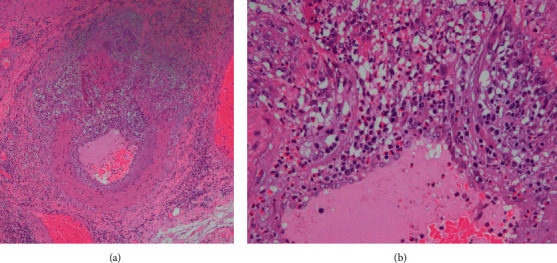
The histopathological examination of the gallbladder. It reveals a necrotic vasculitis and granulomatous inflammation with marked eosinophilic infiltration.

The patient's antinuclear antibody (ANA) titer, antineutrophil cytoplasmic antibody (ANCA), and interferon- (IFN-) *γ* release assays were all negative. Additionally, he presented with renal involvement: proteinuria of 1.37 g/day and tubular dysfunction (N-acetyl-*β*-D-glucosaminidase (NAG), 43 U/L and *β*_2_-microglobulin, 7137 ng/mL). The contrast-enhanced computed tomography (CT) showed ethmoid and frontal sinusitis and cholecystitis. Following a definite diagnosis of acute cholecystitis, piperacillin/tazobactam, at a dose of 13.5 g daily, was started on the first day; however, he showed no clinical improvement and was febrile with worsening fatigue. Because he showed little improvement in the clinical symptoms, a laparoscopic cholecystectomy was performed on day 6. The histopathological findings revealed necrotic vasculitis and granulomatous inflammation with marked eosinophilic infiltration (Figure 2).

**Figure 3:**
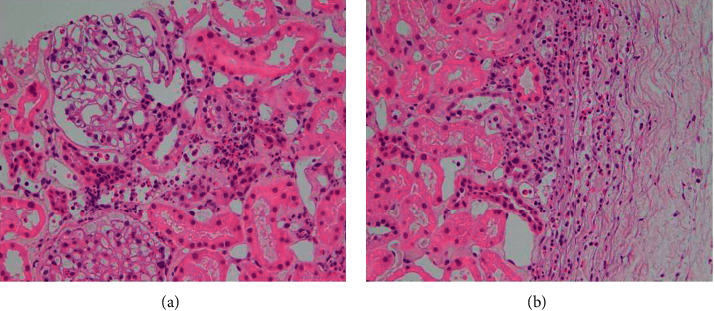
The histopathological findings of the renal biopsy. Marked eosinophil infiltration is found in the interstitium, especially in the subcortex of the kidney, concomitant with tubular atropy. Nephrons are intact.

On day 9, according to these findings, he was diagnosed with EGPA. An endoscopy demonstrated multiple ulcers in the gastric and duodenal antrum. However, an ulcer biopsy did not reveal eosinophilic infiltration or necrotic vasculitis. On day 11, a kidney biopsy was performed, and the kidney tissue examination showed marked eosinophilic infiltration in the tubulointerstitial area and eosinophilic tubulitis, but glomerulonephritis was absent (Figure 3).

Furthermore, a nerve conduction study showed sensory motor mononeuritis multiplex (in the median, peroneal, and tibial nerve, bilaterally). Methylprednisolone (mPSL) pulse therapy (1 g/day for 3 days), followed by oral prednisolone (PSL, 60 mg/day), was started on day 11. However, 7 days after induction therapy, his eosinophil count rose again. Because he showed no response to PSL therapy, his ANCA were negative, and mepolizumab has low side effects, mepolizumab 300 mg was administered on day 18. We started mepolizumab after the approval from the ethics committee (the approval number: H35). His eosinophil count became negative on day 22, and the CRP levels gradually normalized within 2 weeks. On hospitalization day 37, he was discharged with a prescription for a daily dose of PSL 25 mg. Despite rapid PSL reduction, adverse effects or relapse were not observed during hospitalization and after discharge. Monthly mepolizumab therapy had allowed to stop PSL within 4 months.

## 3. Discussion

Here, we have described a case of EGPA, occurring with eosinophilic cholecystitis and interstitial nephritis, successfully treated with induction therapy using mepolizumab.

The present case showed that mepolizumab was effective as induction therapy in an EGPA patient. Typically, PSL is used as first-line induction therapy in EGPA patients; moreover, PSL monotherapy can be used as induction therapy in patients with mild disease [4]. The mPSL pulse therapy or intravenous cyclophosphamide (IVCY) can additionally be used in patients with an FFS ≥2, such as those with severe disease or multiple organ dysfunction. Several studies have reported that treatment with IVCY is associated with higher remission and relapse prevention rates [5]. However, the long-term use of corticosteroids in induction and the use of IVCY are associated with increased risks of adverse reactions, such as infection and cytopenia. Mepolizumab is an interleukin- (IL-) 5 inhibitor approved in Japan for the treatment of EGPA in 2018. IL-5 is essential for the maturation, differentiation, or recruitment of eosinophils; it is involved in the modulation of their activity in allergic inflammation [6]. Moreover, IL-5 plays an important role in the development of EGPA. Wechsler et al. conducted an RCT of mepolizumab compared with a placebo, demonstrating that the drug was effective for maintenance therapy of EPGA and could be useful for tapering steroid doses [3]. However, no studies have reported the use of mepolizumab in induction therapy in EGPA. This is the first study to report the potential usefulness of mepolizumab in induction therapy in patients with severe EGPA.

The pathogenesis of EGPA is related to the two components: eosinophilic inflammation and ANCA. ANCA-positive cases cause severe angiitis, while ANCA-negative ones show more aggressive eosinophilic infiltration [7]. The relationship between eosinophilic inflammation and ANCA titers remains unclear. ANCA induces inflammation in blood vessels by making activated neutrophils release neutrophil extracellular traps (NETs). On the other hand, in many cases of ANCA-negative EGPA, eosinophilic infiltration seems the main etiology because most of those cases are responsive to steroids [8]. Additionally, ANCA-negative cases are linked to larger severe asthma. As mentioned above, IL-5 plays a key role in eosinophilic inflammation. Thus, IL-5 inhibitor mepolizumab is presumably effective to EGPA. Mepolizumab was a good indication in this case which showed ANCA-negative eosinophilia. In terms of clinical presentation based on ANCA status, ANCA-positive patients had a higher incidence of renal involvement, skin involvement, and peripheral neuropathy, while negative ones had more frequent cardiac manifestations [9]. Although ANCA was not positive in this case, interstitial nephritis was observed. This interstitial nephritis is due to eosinophil infiltration, so that was considered a rare case in ANCA-negative patients.

Mepolizumab has an advantage in disease control and safety because it is associated with lower incidence of infusion reactions and adverse reactions than IVCY and rituximab. In the present case, monthly mepolizumab therapy had allowed to stop PSL within 4 months, and no relapse was observed. This suggests that the treatment is also useful in reducing the risk of adverse reactions associated with the long-term use of steroids.

In our case, the diagnosis of EGPA was made after the diagnosis of eosinophilic cholecystitis, which was rare. There are only 16 reported cases of EGPA with eosinophilic cholecystitis. Among these, only one patient presented with interstitial nephritis. Early diagnosis and treatment are important in EGPA patients with rare symptoms, as with the patient in the present case, because diagnosis of this condition tends to be delayed, leading to worse prognosis [10]. In this case, antibiotic treatment was administered for acalculous cholecystitis, which delayed the diagnosis. The findings in this case also suggested that histological examination after surgical resection was useful in the diagnosis; thus, this indicates the importance of including EGPA in the differential diagnosis of patients with multiple organ dysfunction associated with acalculous cholecystitis.

Further accumulation of cases is needed to investigate the effectiveness of mepolizumab as an induction therapy in patients with EGPA, especially those with severe disease.

This study demonstrated that mepolizumab is potentially effective as induction therapy in patients with severe EGPA and can be useful in reducing corticosteroid doses. This was a rare case of EGPA in a patient, occurring concomitantly with eosinophilic cholecystitis and interstitial nephritis. Finally, our experience indicates that clinicians should be aware of the possibility of EGPA when a patient presents with multiple organ dysfunction associated with eosinophilia.

## Figures and Tables

**Table 1:** Results of the complete blood count, biochemistry, and urinalysis at admission.

Hematology	
White blood cells	14300/*μ*L
Eosinophils	8000/*μ*L
Red blood cells	432 × 10^4^/*μ*L
Hemoglobin	14.1 g/dL
Platelet	29.0 × 10^4^/*μ*L
Biochemistry	
Total protein	7.7 g/dL
Albumin	2.9 g/dL
AST	50 IU/L
ALT	94 IU/L
LDH	310 IU/L
Total bilirubin	0.5 mg/dL
Creatinine	0.96 mg/dL
Blood urea nitrogen	11 mg/dL
Sodium	136 mmol/L
Potassium	3.8 mmol/L
Chloride	100 mmol/L
C-reactive protein	7.4 mg/dL
sIL-2R	3077 IU/mL
Infection	
Interferon-*γ* release assay	Negative
HIV Ab	Negative
Immunology	
Immunoglobulin G	2209 mg/dL
Immunoglobulin G4	1220 mg/dL
Immunoglobulin A	97 mg/dL
Immunoglobulin M	87 mg/dL
Immunoglobulin E	2482 IU/mL
Rheumatoid factor	123 IU/mL
Antinuclear Ab	<40×
Anticardiolipin Ab	<8 IU/mL
MPO-ANCA	<1.0 IU/mL
PR3-ANCA	<1.0 IU/mL
Anti-GBM Ab	<2.0 IU/mL
C3	103 mg/dL
C4	24 mg/dL
Urinary	
pH	7.5
Specific gravity	1.020
Urine protein	1.37 g/gCre
Red blood cells	1/HPF
White blood cells	1–4/HPF
*β*_2_-microglobulin	7137 *μ*g/L
NAG	43 IU/L

Ab, antibody; ALT, alanine aminotransferase; AST, aspartate aminotransferase; GBM, glomerular basement membrane; HIV, human immunodeficiency virus; HPF, high power field; IU, international unit; LDH, lactate dehydrogenase; MPO-ANCA, myeloperoxidase-antineutrophil cytoplasmic antibody; NAG, N-acetyl-*β*-D-glucosaminidase; PR3-ANCA, proteinase-3-antineutrophil cytoplasmic antibody; sIL-2R, soluble interleukin-2 receptor antibody.

## Data Availability

The data used to support the findings of this study are included within the article.
